# Unraveling the complex genetic basis of growth in New Zealand silver trevally (*Pseudocaranx georgianus*)

**DOI:** 10.1093/g3journal/jkac016

**Published:** 2022-01-20

**Authors:** Noemie Valenza-Troubat, Sara Montanari, Peter Ritchie, Maren Wellenreuther

**Affiliations:** 1 Seafood Production Group, The New Zealand Institute for Plant & Food Research Ltd, Nelson 7010, New Zealand; 2 School of Biological Sciences, Victoria University of Wellington, Wellington 6140, New Zealand; 3 School of Biological Sciences, The University of Auckland, Auckland 1010, New Zealand

**Keywords:** linkage map, QTL, genome-wide association studies, selective breeding, genomic prediction, GenPred, shared data resource

## Abstract

Growth directly influences production rate and therefore is one of the most important and well-studied traits in animal breeding. However, understanding the genetic basis of growth has been hindered by its typically complex polygenic architecture. Here, we performed quantitative trait locus mapping and genome-wide association studies for 10 growth traits that were observed over 2 years in 1,100 F_1_ captive-bred trevally (*Pseudocaranx georgianus*). We constructed the first high-density linkage map for trevally, which included 19,861 single nucleotide polymorphism markers, and discovered 8 quantitative trait loci for height, length, and weight on linkage groups 3, 14, and 18. Using genome-wide association studies, we further identified 113 single nucleotide polymorphism-trait associations, uncovering 10 genetic hot spots involved in growth. Two of the markers found in the genome-wide association studies colocated with the quantitative trait loci previously mentioned, demonstrating that combining quantitative trait locus mapping and genome-wide association studies represents a powerful approach for the identification and validation of loci controlling complex traits. This is the first study of its kind for trevally. Our findings provide important insights into the genetic architecture of growth in this species and supply a basis for fine mapping quantitative trait loci, genomic selection, and further detailed functional analysis of the genes underlying growth in trevally.

## Introduction

Growth facilitates essential functions such as reproduction or the ability to adapt to environments. Although there are exceptions, increase in body size is usually positively correlated with numerous fitness traits such as higher mating success and fecundity, increased offspring quality and lengthened longevity (Gebhardt-Henrich and Richner 1998; Dmitriew 2011). Most vertebrates exhibit a finite amount of growth. In fish, however, the process of growing is indeterminate, or indefinite, and continues throughout their life (Weatherley 1972), although the rate tends to decline as body size increases (Pedersen and Jobling 1989). This highly complex process is the result of interactions between environmental effects and genetic differences. Factors including sex (e.g. Imsland and Jonassen 2003), age, or food availability (e.g. Jones 1986) influence growth rate, as well as abiotic factors changing seasonally such as temperature (e.g. Karås and Klingsheim 1997; Imsland *et al.* 2007), photoperiod (e.g. Imsland and Jonassen 2003), or oxygen levels (e.g. Brett and Groves 1979). The genetic basis of growth traits is typically highly polygenic (Wellenreuther and Hansson 2016). Functional relationships between genetic variations and physiological parameters of growth have been described in commercially relevant species such as cod (*Gadus morhua*) [see review by Imsland and Jónsdóttir (2002)], Atlantic salmon (*Salmo salar*) (Tsai *et al.* 2015), or tilapia (*Tilapia mossambica*) (Liu *et al.* 2014), but are still poorly understood in many nonmodel species.

With the development of next-generation sequencing technologies the cost of high-density genotyping has drastically decreased, therefore enabling the more widespread application of quantitative trait locus (QTL) mapping experiments. Many variants associated with complex phenotypes have since been found in a variety of nonmodel species. However, QTL mapping approaches only allow the identification of genetic regions that are polymorphic between 2 parents, and relevant in a particular environment, therefore, potentially missing some common variants associated with the traits (Mackay 2001). For this reason, the location and effects of detected QTLs can vary between mapping populations. For example, in the Atlantic salmon, QTLs involved in growth have been found on different linkage groups (LGs) according to different studies (Baranski *et al.* 2010; Gutierrez *et al.* 2012; Tsai *et al.* 2015; Besnier *et al.* 2020).

Recently, genome-wide association studies (GWAS) have been widely employed to detect QTLs both in captive (Palaiokostas *et al.* 2018) and natural populations (Santure and Garant 2018). This technique identifies associations between markers and phenotypes based on linkage disequilibrium (LD) (Meuwissen and Goddard 2010). Compared with QTL mapping, GWAS can be conducted on genetically diverse, unrelated individuals and is particularly advantageous when controlled crossing and generation of large segregating populations is difficult. GWAS has proved useful for the identification of loci associated with numerous growth traits in fish (e.g. Yang *et al.* 2020). However, the power of GWAS relies on the number of markers in relation to the extent of LD in the population (Newell *et al.* 2011). When applied to natural populations or collections of outcrossing individuals, characterized by a rapid LD decay, tens of thousands of markers are often required to obtain an adequate level of resolution. Because of the intrinsic limitations of statistical methods comparing thousands of tests, GWAS has a higher frequency of false discovery of loci than QTL mapping (Hayes *et al.* 2013). A final disadvantage of GWAS is its low power for detecting rare allelic variants. However, their frequency can be increased in controlled crosses and therefore captured with QTL mapping. Hence, combining GWAS and QTL mapping often gives more complete and reliable results than using one of the methods alone (Fraslin *et al.* 2020).

Aquaculture is the fastest growing food-production sector in Aotearoa New Zealand. Currently, the local industry relies almost exclusively on the farming of 3 species: Greenshell mussels (*Perna canaliculus*), Pacific oysters (*Crassostrea gigas*), and only 1 finfish, king/chinook salmon (*Oncorhynchus tshawytscha*), which is an introduced species (Camara and Symonds 2014; Symonds *et al.* 2019). Hence, there is a strong interest in diversifying the range of species available for aquaculture. The native finfish silver trevally (*Pseudocaranx georgianus*, Cuvier 1833) has been identified as a suitable candidate for New Zealand aquaculture. In Aotearoa, its Māori name is araara. Indigenous Māori people have a strong cultural connection to trevally, where it is considered as taonga (i.e. has value, or is treasured). Trevally is a shoaling pelagic species found throughout the coastal waters of southern Australia and around New Zealand (Smith-Vaniz and Jelks 2006). It is most common at depths of approximately 80 m, although its range is thought to be 10–238 m and can reach over 40 years of age (Ministry for Primary Industries 2014; Bray 2020). In many regions of New Zealand, trevally is a major component of recreational and commercial fisheries (Ministry for Primary Industries 2014). For it to be suitable for commercial aquaculture, however, trevally’s growth rate must be improved, which can be done using selective breeding (Valenza-Troubat *et al.* 2021b).

In 2016, the Institute of Plant and Food Research (PFR) started a selective breeding program on trevally, and has induced a wild broodstock to create F_1_ offspring for this purpose. The primary goal of the breeding program is to improve the growth rate, which has been demonstrated to show both high heritability and transcriptional plasticity in response to temperature (Valenza-Troubat *et al.* 2021a, 2021b). Individuals attain sexual maturity between years 3 and 4, which is also around the time when this species reaches harvest size (Valenza-Troubat *et al.* 2021b). The long-term breeding goal is to reduce the time to harvest to around 2 years. In this study, we investigated the genetic architecture of 10 growth traits in the F_1_ new population of trevally. More specifically, we genotyped and phenotyped 1,100 F_1_ trevally to (1) generate a high-density linkage map; (2) detect rare QTLs associated with growth using linkage mapping in a subfamily comprising 89 individuals; (3) identify common SNPs associated with growth using GWAS on the whole population; and (4) overlap the results and annotate supported regions.

## Methods

### Study population

The trevally population used in this study was generated as part of a breeding program started at the PFR finfish research facility in Nelson, New Zealand. The full description of holding conditions and the pedigree are described in Valenza-Troubat *et al.* (2021a, 2021b). Briefly, the population comprised of 13 wild caught F_0_ broodstock and 1,100 F_1_ captive-bred offspring. In 2015, induced mass spawning of the F_0_ generation in a single tank was used to produce the offspring F_1_ generation. This resulted in a complex pedigree, including a combination of unrelated, full-, and half-siblings in the F_1_ generation. F_1_ offspring were held in a single tank from hatch receiving the same feeding regime, light, water flow, and aeration until the end of this experiment. The seawater tanks in the facility are located on the seaward side of Port Nelson and receive ambient seawater from an underground bore that is filtered using mesh filters and UV treatment.

### Phenotyping and trait estimation

Ten growth traits were used in the current study, namely peduncle length (PL), height at 25 (H25), 50 (H50), and 75% (H75) of the PL, estimated weight (EW), and related net gain traits (ΔPL, ΔH25, ΔH50, ΔH75, and ΔEW, respectively) (Valenza-Troubat *et al.* 2021b). These measurements were recorded on 3 occasions throughout the experiment, at the beginning (November 2017), in the middle (October 2018), and at the end (November 2019), when the fish were a little over 2, 3, and 4 years old, respectively. Using the Morphometric Software (https://www.plantandfood.co.nz/page/morphometric-software-home/), the outline of each fish was extracted from images and morphometric were located, and then used to make measurements. PL was measured by assessing the distance between the upper lip and narrowest cross-section of the tail. Height was measured at 3 positions along the fish: 25%, 50%, and 75% of the PL. The weight estimations (EW) were done following a Bayesian hierarchical approach as described in Froese *et al.* (2014). The net gain for each trait was calculated as the difference from the initial measurement (November 2017) with subsequent ones. The normality of the data was assessed visually using quantile–quantile (QQ) plots generated in the R statistical environment version 3.2.3 (R Development Core Team 2016).

### Genotyping and variant calling

Samples of fin tissue from 13 tagged F_0_ and 1,100 F_1_ individuals were collected and stored as described in Valenza-Troubat *et al.* (2021a, 2021b). Total DNA was extracted as described in Ashton *et al.* (2019) with minor modifications, and then quantified and quality-checked by fluorescence, spectrophotometry and agarose gel electrophoresis. The 13 F_0_ individuals were whole genome sequenced (paired-end, 125 bp reads) over 3 lanes of the HiSeq 2500 platform at the Australian Genome Research Facility (AGRF, Melbourne, Australia). The F_1_ were genotyped using a modified GBS approach (Elshire *et al.* 2011), as described in Valenza-Troubat *et al.* (2021a, 2021b). A total of 12 pools of 96 samples each were prepared and sent to AGRF for sequencing on a HiSeq 2500 platform (single-end, 100 bp reads). Sequencing data quality for both F_0_ and F_1_ generations were checked using FastQC v0.11.7 (Andrews 2010). Raw reads from the F_0_ were trimmed using trimmomatic v0.36 (Bolger *et al.* 2014) (using the parameters HEADCROP: 9; TRAILING: 10; SLIDINGWINDOW: 5:20; MINLEN: 75). The F_1_ samples were demultiplexed from the 12 sequencing libraries using the process_radtags module available in the STACKs version 2.1 pipeline (Catchen *et al.* 2013) and the reads were trimmed using Fastq-mcf in ea-utils v1.1.2-806 (minimum sequence length = 50, quality threshold causing base removal = 33) (Aronesty 2013). Read groups were added to all sequences and bam files were sorted and indexed using Picard toolkit (Broad_Institute 2015). Reads were then mapped to the reference genome (Catanach *et al.* 2021) using the Burrows–Wheeler Aligner v0.7.17 (Li and Durbin 2010) and variants were called jointly with the parallel module of freebayes v1.3.1 (Garrison and Marth 2012), with minimum of 5 observations and minimum mapping quality of 10. The SNPs with single-sample sequence coverage (sequencing depth) < 3 were removed to reduce the number of putatively erroneous genetic variants, and missing data and minor allele frequency (MAF) were set to <20% and >0.05, respectively.

### Linkage map construction and QTL identification

The parents of each F_1_ individual in the dataset were identified with Sequoia v2.0.7 (Huisman 2017) as reported in Valenza-Troubat *et al.* (2021a, 2021b). The full SNP dataset (i.e. before the stringent filtering performed for the parentage analysis) was filtered for Mendelian errors (>5%), and checked for distorted segregation using a chi-square test with α = 0.05. The linkage map was constructed in Lep-MAP v3.0 (Rastas *et al.* 2013), using the largest family. Markers were separated into linkage groups (LGs) with the SeparateChromosomes module [logarithm of odds (LOD) limit = 14, minimum markers per LG = 50]. The order of the markers was computed with the OrderMarkers module. Single markers at the end of each LG were removed if they were more than 3 cM apart from the next closest marker. MapChart v2.32 (Voorrips 2002) was used to visualize the genetic map. As the F_0_ were assumed to be outbred, the linkage map and the genotypes of the mapping family were input as a 4-way cross in the R package R/qtl version 1.47-9 (Broman *et al.* 2003) for interval mapping. Standard interval mapping was performed and a genome-wide permutation test (Doerge and Churchill 1996) with 1,000 permutations was used to determine the LOD significance thresholds (*P*-value = 0.05).

### Genome-wide association study

GWAS was carried out on the entire genotyped F_1_ population (*n* = 1,100). SNPs were removed if the call rate was smaller than 0.8, MAF < 0.01, if Mendel error rate >5% (based on trios identified with the parentage analysis carried out above), and they were LD-pruned using an *r*^2^ > 0.80 in a 50-kb sliding window with 5 variants. Association analysis were performed using the Fixed and random model Circulating Probability Unification (FarmCPU) method (Liu *et al.* 2016) implemented in GAPIT3 v3.1.0 (Wang and Zhang 2021). A Bayesian information criterion (BIC)-based model selection was used to find the optimal number of principal components (PCs) for each time measure, to account for family and population structure. The cutoff for significant association was a false discovery rate (FDR)-adjusted *P*-value = 0.05 (Benjamini and Hochberg 1995), to control for multiple testing. To assess how well the model used in GWAS accounted for population structure and family relatedness, results of the GWAS were visualized with QQ plots implemented in GAPIT3, which depicted the distribution of the actual *P*-values compared with the theoretical ones. Manhattan plots were used to visualize the SNPs associated with the different phenotypes, using the physical position of the markers on the reference genome.

### Ethics statement

All research carried out in this study was reviewed and approved by the animal ethics committee of Victoria University of Wellington in New Zealand (application number 25976).

## Results

### Phenotypic values of growth traits

**Fig. 1.**
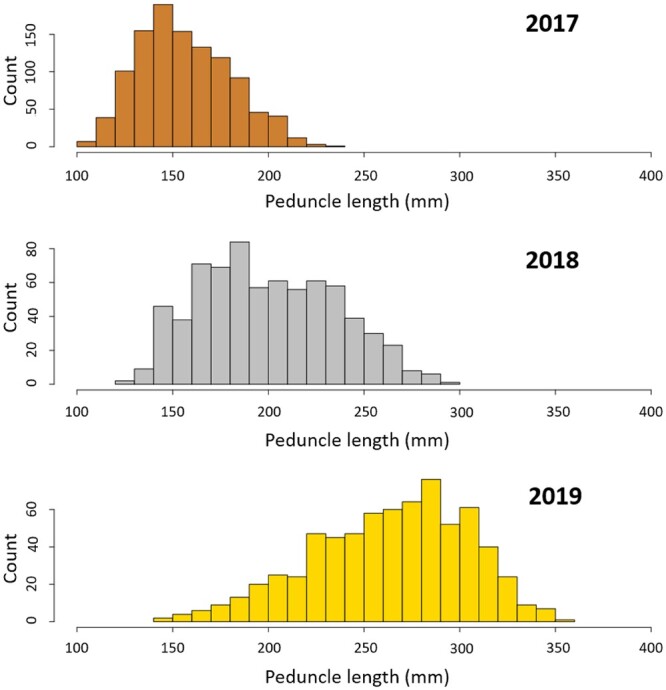
Distribution of the PL measurements in November 2017 (*n* = 1,094), October 2018 (*n* = 719), and November 2019 (*n* = 694).

All offspring were phenotyped at the first measurement (November 2017), while numbers decreased at the 2 subsequent time points because of natural mortality that occurred during the study. All traits showed a normal distribution and exhibited large levels of phenotypic variation, as discussed in Valenza-Troubat *et al.* (2021a, 2021b). A summary of the mean values and standard deviations of the 10 growth phenotypes is shown in Table 1. In both the family used for QTL mapping and in the entire population used for GWAS, a normal distribution of the residuals was observed for the 10 traits investigated (Fig. 1). Transgressive lines (i.e. offspring that have more extreme phenotypes than the parents) were observed in the population.

**Table 1.** Summary of the phenotypes height at 25%, 50%, and 75% of the body (H25, H50, and H75, respectively), PL, EW, and net gains traits associated (ΔH25, ΔH50, ΔH74, ΔPL, and ΔEW, respectively) across the whole F_1_ population and for the largest family.

	November 2017		October 2018		November 2019	
All	*n* = 1,094		*n* = 719		*n* = 694	
	Mean	SD	Mean	SD	Mean	SD
H25	49.71	9.02	66.94	13.51	90.10	15.70
H50	59.45	11.02	79.99	16.39	105.53	18.65
H75	48.58	9.91	61.26	12.69	85.63	15.80
PL	156.79	23.92	200.64	35.36	264.09	40.44
EW	89.90	41.85	191.93	99.89	426.34	177.93
ΔH25			15.85	7.80	39.08	11.21
ΔH50			18.75	8.42	44.34	12.36
ΔH75			11.08	6.77	35.54	10.19
ΔPL			40.03	18.00	103.59	27.31
ΔEW			95.83	64.45	330.78	147.12

Family	***n* = 87**		***n* = 68**		***n* = 67**	
	**Mean**	**SD**	**Mean**	**SD**	**Mean**	**SD**

H25	52.21	7.95	73.71	11.32	96.52	12.64
H50	63.10	9.29	86.87	13.41	111.90	14.77
H75	52.85	8.99	66.72	9.75	92.36	12.39
PL	167.59	21.32	218.19	30.04	280.78	33.07
EW	107.43	37.85	238.01	89.61	498.52	159.82
ΔH25			20.43	6.53	42.78	9.21
ΔH50			22.46	6.92	46.84	9.89
ΔH75			12.88	5.54	37.79	8.33
ΔPL			47.91	15.91	109.31	23.15
ΔEW			125.99	60.54	384.63	135.00

Included are the number offspring phenotyped (*n*), mean, and SD.

### High-density linkage map

**Fig. 2.**
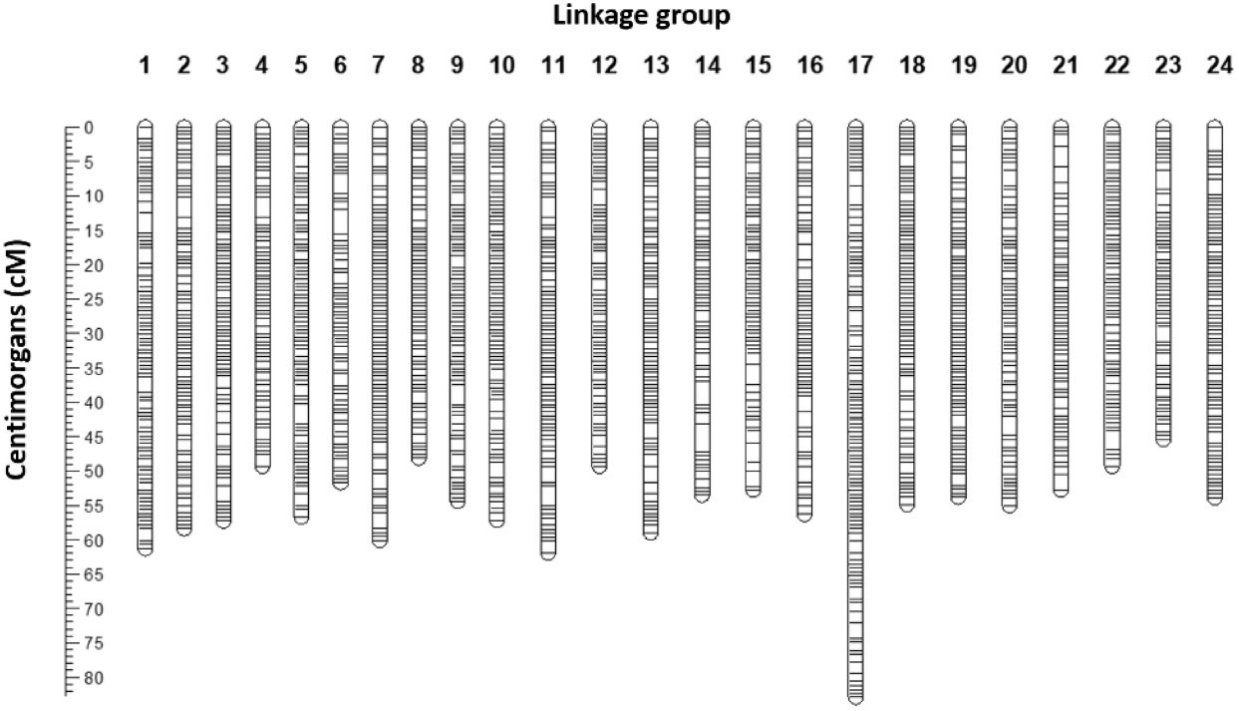
Visualization of the sex-averaged map built for the largest segregating family (*n* = 87). The 24 LGs represent the expected 24 *Pseudocaranx georgianus* chromosomes.

Sequencing of the 13 parental fishes generated 1.23 billion reads, corresponding to an average genome coverage of 13× per individual. Of the 1,100 offspring, 1,094 F_1_ were successfully genotyped via GBS, resulting in 0.42× genome coverage per individual fish. Variant calling and basic quality filtering generated a dataset of 171,923 SNPs. Overall, 21 families from 10 out of the 13 sequenced F0 fishes were identified by Valenza-Troubat *et al.* (2021a, 2021b) when reconstructing the pedigree of the population. The largest family included 87 offspring and was used to assemble the sex-averaged linkage map. A total of 21,665 SNPs were polymorphic in this family and passed the chi-square test, and 19,861 were successfully mapped to 24 LGs, amounting to 1,830 when not accounting for the comapping loci (Fig. 2). Inspection of SNPs showed that there was good congruence between the physical location of SNPs on the map vs the genome. LG numbers were randomly assigned, as no previous reference had been published. The genetic map spanned 1,335.46 cM, with an average marker distance of 0.73 cM. The largest gap was on LG14 and was 4.10 cM long. The longest and shorter LGs were 17 (82.89 cM) and 8 (48.14 cM), respectively (Table 2).

**Table 2.** Marker statistics of the linkage map constructed from the largest trevally family.

LG	Number of markers^a^	Length (cM)	Average distance between markers (cM)
1	85	61.27	0.72
2	80	58.41	0.73
3	81	57.26	0.71
4	68	49.34	0.73
5	81	56.69	0.70
6	65	51.72	0.80
7	87	60.09	0.69
8	70	48.14	0.69
9	78	54.43	0.70
10	84	57.22	0.68
11	82	61.88	0.75
12	71	49.30	0.69
13	79	59.00	0.75
14	64	53.51	0.84
15	67	52.84	0.79
16	67	56.27	0.84
17	108	82.89	0.77
18	82	54.95	0.67
19	80	53.82	0.67
20	71	55.04	0.78
21	65	52.83	0.81
22	73	49.29	0.68
23	63	45.39	0.72
24	79	53.88	0.68
Total	1,830	1.335.46	0.73

a Count after removal of comapping.

### QTL mapping revealed rare variants associated with growth

**Fig. 3.**
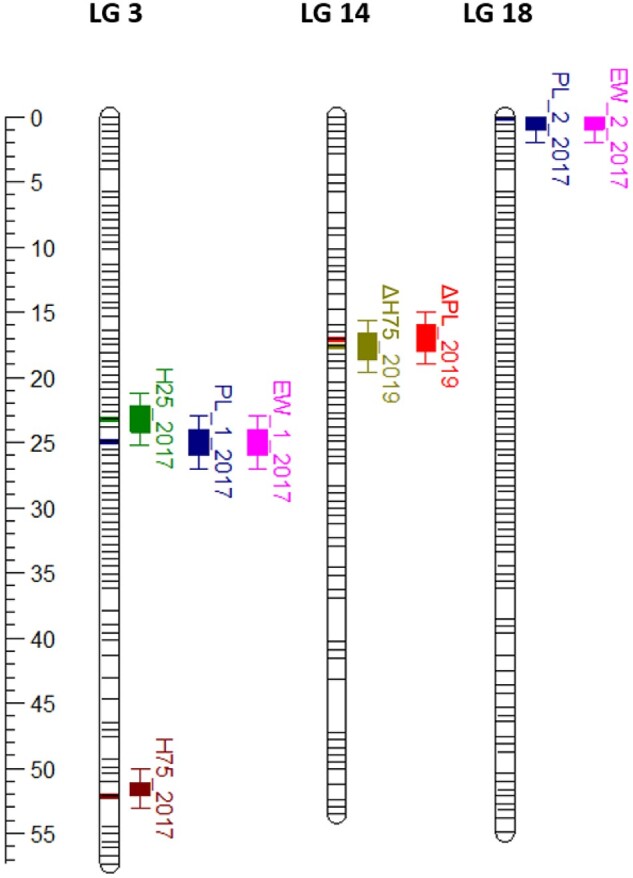
Eight significant QTL associated with growth traits were found in trevally, across 3 LGs (LG3, LG14, and LG18). In November 2017, 2 QTLs were found associated with peduncle length (PL_1_2017 and PL_2_2017, on LG3 and LG18, respectively), which were also associated with EW (EW_1_2017 and EW_2_2017). In the same year, 2 more QTLs were found associated with height at 25% (H75_2017) and 75% (H75_2017) of the peduncle length, both on LG3. Finally, 2 QTLs were found in November 2019, 1 for net gain in peduncle length (PL_2019) and one for net gain in height at 75% of the peduncle length (H75_2019), both on LG14.

The genome-wide significant thresholds for QTL mapping were established at LOD values between 4.60 and 4.81 for the 10 phenotypic traits across 3 years (Table 3). In total, 8 significant QTLs were identified on 4 locations in 3 LGs (Fig. 3). Six of these QTLs were for traits recorded during the first measurement (November 2017), and 2 for the last measurement (November 2019). In particular, 3 QTLs detected for H25, PL, and EW measured at the first time point comapped on LG3 (between 23.2 and 24.9 cM). A second QTL was mapped to LG3 at 52.1 cM for H75 from the first measurement. Additionally, the traits PL and EW from 2017 were associated to a second locus, at the top of LG18. The percentage of phenotypic variance explained (PVE) by the 2017 QTLs ranged from 22.1% to 25.3%. In the last measurement, 2 QTLs for ΔH75 and ΔPL were found to comap to LG14 (between 17 and 17.1 cM), with PVE of 39.7 and 31.1, respectively (Table 3). No QTLs were found for the middle measurement (October 2018) and for all other traits in 2017 and 2019.

**Table 3.** List of significant QTL detected for height at 25% and 75% of the peduncle length (H25 and H75, respectively), PL, EW, and net gain for H75 (ΔH75) and PL (ΔPL) in November 2017 (Nov17) and November 2019 (Nov19), using a high-density linkage map.

Time	Trait	LG	Peak position (cM)	SNP at peak	LOD threshold	LOD at peak	PVE (%)
Nov17	H25	3	23.2	trevally000114_4454829	4.71	4.73	22.1
Nov17	PL	3	24.9	trevally000114_4498123	4.71	4.83	22.6
Nov17	EW	3	24.9	trevally000114_4498123	4.60	5.39	24.8
Nov17	H75	3	52.1	trevally000114_23355470	4.64	5.50	25.3
Nov17	PL	18	0.0	trevally001200_2242455	4.71	4.88	22.8
Nov17	EW	18	0.0	trevally001200_2242455	4.60	4.82	22.5
Nov19	ΔPL	14	17.0	trevally001025_25205374	4.81	5.41	31.0
Nov19	ΔH75	14	17.6	trevally001025_25180484	4.69	7.36	39.7

For each QTL the position on the LG, the significance threshold, the LOD at the peak, the SNP name, and the PVE are shown.

### Genome-wide association found strongly associated SNPs

**Fig. 4.**
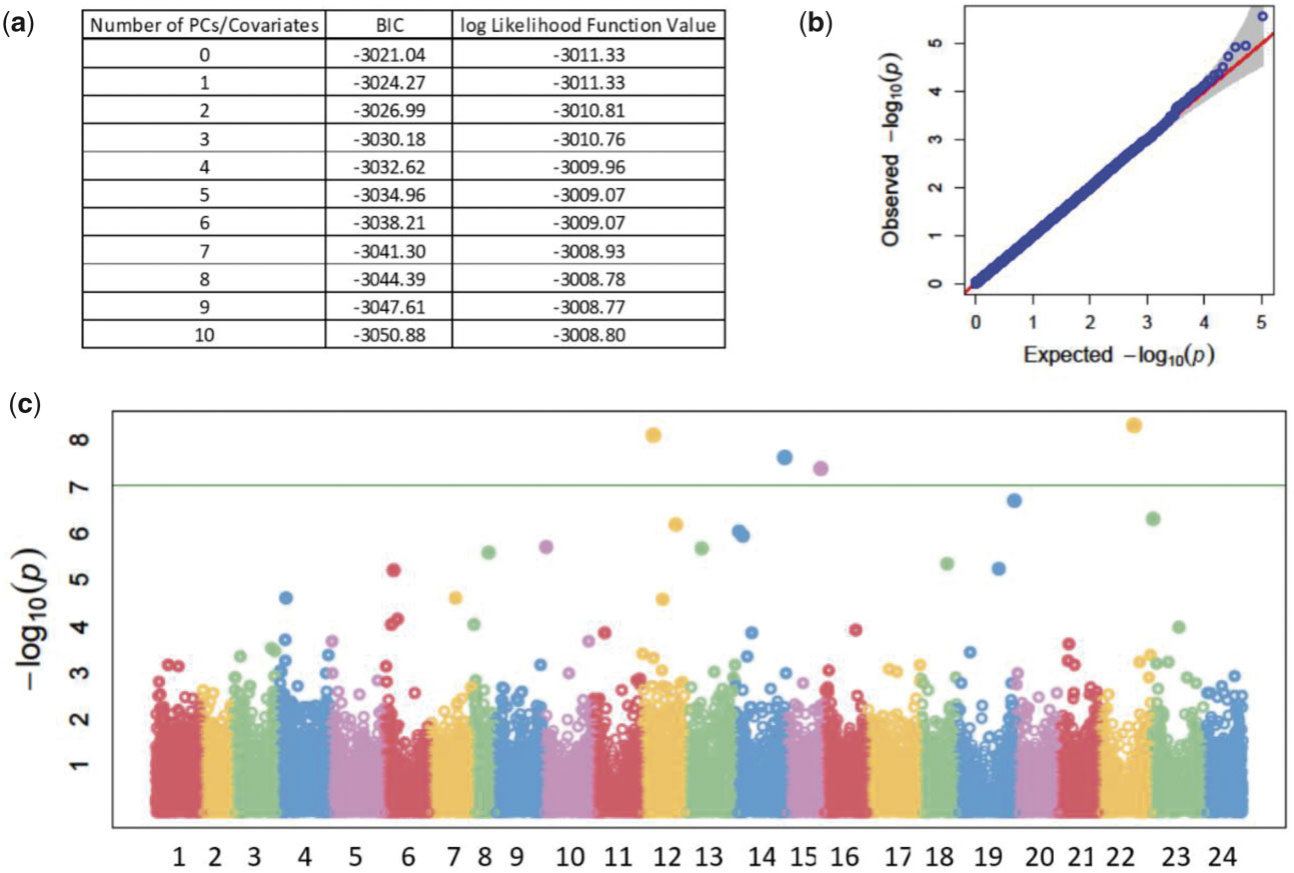
GWAS results for the net gain in PL (ΔPL) in November 2019. a) Summary of BIC for the optimal number of PCs to use in the model. b) QQ-plot of *P*-values. On the *Y*-axis is the observed negative base 10 logarithm of the *P*-values, and on the *X*-axis the expected observed negative base 10 logarithm of the *P*-values under the assumption that the *P*-values follow a uniform [0 to 1] distribution. The gray area shows the 95% confidence interval for the QQ-plot under the null hypothesis of no association between the SNP and the trait. c) Manhattan plot of the results of the GWAS. On the *X*-axis is the physical position of the SNPs on the genome divided by chromosomes, and on the *Y*-axis is the negative log base 10 of the *P*-values. The horizontal green line represents the significance threshold.

A total of 1,024 F_1_ individuals had both genotypic and phenotypic data for all measurements. After filtering based on Mendelian errors, MAF, and LD pruning, 107,067 SNPs were left and used in the GWAS. Model selection in GAPIT resulted in no PCs to be used as covariates in any of the traits (Fig. 4a). QQ plots showed that FarmCPU adequately accounted for the confounding effects of family and population structure (Fig. 4b). A total of 93 SNPs were significantly associated [−log 10(ρ) > 7.03] with at least 1 of the 10 traits measured at each year of phenotyping (Fig. 4c;Supplementary Table 1). Only ΔH75 in October 2018 and November 2019 had no significant association. Of the 93 SNPs, 15 were associated with more than 1 trait. These included 4, 7, and 4 SNPs associated with measurements at the first, second, and third time point, respectively. No common SNPs were identified among different years. Significant associations were in some cases found for SNPs <0.5 Mb apart, highlighting hot spots on chromosomic regions. A total of 10 hot spots were identified: 2 on LG1, 1 on LG2, 2 on LG3, 2 on LG5, 1 on LG6, 1 on LG10, and 1 on LG18. Six of these included associations for traits measured in different years (Supplementary Table 1).

## Discussion

We constructed the first genetic map for trevally, and we were able to use this map to determine genomic regions associated with phenotypic growth traits. Our map included 19,861 SNPs across 24 LGs and confirmed the 24 mega-scaffolds of the trevally reference genome (Catanach *et al.* 2021). A linkage map built for yellowtail kingfish (*Seriola lalandi*), the closest species to trevally for which a map has been constructed, was also resolved into 24 LGs (Nguyen *et al.* 2018), consistent with our findings. The map built here for trevally was 1,335.46 cM in length, which is within the range of map length expected for many teleost fish. For example, the map assembled here is longer when compared with the yellowtail kingfish map, similar in length to the map of the Australasian snapper (*Chrysophrys auratus*) (Ashton *et al.* 2019) and shorter when compared with the European sea bass (*Dicentrarchus labrax*) (Griot *et al.* 2021).

Eight significant QTLs related to growth were mapped to 3 of the trevally LGs. Other studies in teleost have found multiple QTLs affecting growth such as in Atlantic salmon (Tsai *et al.* 2015; Besnier *et al.* 2020), Australasian snapper (Ashton *et al.* 2019), spotted sea bass (*Lateolabrax maculatus*) (Liu *et al.* 2020), or yellowtail kingfish (Nguyen *et al.* 2018), supporting the hypothesis of a polygenic regulation. No significant QTLs were identified with QTL mapping for the second time point, October 2018. In the segregating family used for QTL mapping, the number of offspring decreased from 87 in 2017 to 68 in 2018 and 67 in 2019 as the result of natural mortality. Although some statistically significant QTLs were found in the measurements made in the latter period of the experiments, the smaller sample sizes in the 2 last measurements reduced the power of the study, which could explain the absence of QTLs detected in October 2018. Indeed, sample size is known to influence the power of a study to detect QTLs (Hong and Park 2012) and is regularly discussed as one of the most important concerns when designing a mapping experiment (Ashton *et al.* 2017). For the same reason, QTL mapping was not performed on the smaller families present in this breeding population. Interestingly, 2 traits (PL and EW) shared the same QTLs on 2 LGs in the first measurement period (November 2017). This is consistent with the level of high genetic and phenotypic correlations reported for body length and body weight (Valenza-Troubat *et al.* 2021b). These findings suggest that selection applied on easily measurable traits such as length will result in the concomitant improvement of more difficult to assess, yet valuable, growth traits (such as weight) in the breeding of trevally.

GWAS identified 113 associations with growth, corresponding to 93 different SNPs spanning 22 LGs, further supporting the hypothesis of a polygenic basis of growth-related traits in trevally. Growth is considered as a complex trait and has been found to be polygenic across the tree of life, in very diverse taxa from plants, like in the model species *Arabidopsis thaliana* (Wieters *et al.* 2021), to vertebrates like humans (Sinnott-Armstrong *et al.* 2021) and other fish species (e.g. Liu *et al.* 2014; Yang *et al.* 2020; Debes *et al.* 2021). In our study, a lower number of loci were found with the QTL mapping experiment than with the GWAS, which can be explained by the smaller amount of genetic variation represented in the single F_1_ family compared with the overall breeding population, which was derived from 13 parental individuals. Four QTLs and 15 SNPs were found to have a significant association with more than 1 trait. This was expected, as the different phenotypes were all deduced from the PL and they all then measured the same process underlying growth.

**Fig. 5.**
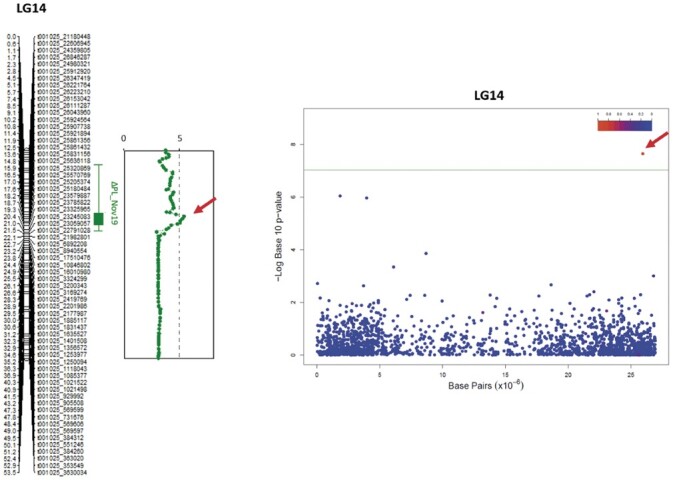
Comparison of QTL (left) and SNP associated (right) with the net gain in PL (ΔPL) in November 2019.

For each trait, the markers found in association were different from a year to another, both in QTL mapping and in GWAS. These differences could be due either to significant loci changing over time, by switching on or off, or to environmental variation, as it was observed in other QTL mapping studies that investigated traits highly affected by the environment (Sun *et al.* 2017; Ashton *et al.* 2019). However, there were some hot spot regions of 0.5 Mb that contained SNPs associated with different traits and different years. Noteworthy is the chromosomic region at the top of LG5, where 2 hot spots were found, 1 between 0 and 0.5 Mb (for ΔEW and PL in 2018, and H75 and ES in 2019) and one between 1.4 and 1.8 Mb (for ΔEW, H25, H50, and PL in 2019). Future studies should investigate these further. In addition to these hot spots, we also identified SNP regions that were significant in both QTL and GWAS analyses. Specifically, by comparing the relative physical positions of the SNPs, 2 regions found with QTL mapping appeared to be in close proximity with 2 significant SNPs identified by GWAS. In particular, the QTLs for H25, PL, and EW measured in 2017 spanned the 4.45- to 4.50-Mb region on chromosome 3 (corresponding to 23.20–24.90 cM on LG3) and SNP trevally000114_4956408, located at 4.96 Mb on chromosome 3, was found to be significant for PL in 2019 with GWAS; and the QTLs for ΔH75 and ΔPL in 2019, encompassing the 25.18–25.21 Mb region on chromosome 14 (17.02–17.59 on LG14), are close to SNP trevally001025_25939455, located at 25.94 Mb on chromosome 14 and significant for ΔH50 and ΔPL in 2019 (Fig. 5 and Table 3; Supplementary Table 1). Being identified with 2 different statistical analyses and for multiple traits across 2 years, these 2 regions are then of particular interest for the understanding of the genetic determinism of growth in trevally. A BLAST search of the 100-kb regions flanking those markers against the NCBI nucleotide database did not return any sequence similarities, indicating that they are located in noncoding or in nonannotated regions. Intergenic regions can still have a functional role in gene expression and regulation (Wyrick and Young 2002). For example, these SNPs could be located in an intron that acts as a regulatory region (promoter, enhancer, silencer, or insulator).

Compared with the heritability estimates found in Valenza-Troubat *et al.* (2021a, 2021b), the present analysis still seems underpowered. The range of heritability estimates was moderate to high (0.67 ± 0.05 to 0.76 ± 0.06) for the measured traits (H25, H50, H75, PL, and EW) and moderate (ranging from 0.28 ± 0.07 to 0.68 ± 0.07) for the net gain traits (ΔH25, ΔH50, ΔH75, ΔPL, and ΔEW), and it remained consistent throughout the experiment. In the present study, PVEs ranged from 22.1% to 47.36% for the QTL mapping study and from 4.74% to 46.81% for GWAS, indicating that neither techniques were able to capture all of the genetic components of growth traits (Table 4). Additional genetic interactions, other than additive effects, should be considered. While heritability is a key feature of a trait indicating its potential for improvement via selection, polygenic traits are often influenced by nonadditive genetic effects such as dominance or epistasis (Glover *et al.* 2017). Understanding the genetic mechanisms that underlie a trait is an important part of explaining phenotypic diversity. This is particularly relevant when looking at traits related to fitness (e.g. growth, shyness, foraging, or predator awareness) in populations that are undergoing domestication but still occasionally interbreed with wild conspecifics (e.g. when new broodstock is caught and added).

**Table 4.** Comparison of heritability estimates found in Valenza-Troubat *et al.* (2021a, 2021b) and PVE by QTL found in interval mapping and markers from the GWAS.

Time	Trait	Heritability	PVE QTL (%)	PVE GWAS (%)
Nov17	H25	0.67 ± 0.05	22.1	18.4
Nov17	H50	0.75 ± 0.05	—	24.2
Nov17	H75	0.73 ± 0.05	25.3	38.5
Nov17	PL	0.74 ± 0.05	45.3	28.8
Nov17	EW	0.75 ± 0.05	47.4	42.3
Oct18	H25	0.74 ± 0.05	—	38.6
Oct18	H50	0.75 ± 0.05	—	24.2
Oct18	H75	0.68 ± 0.05	—	4.7
Oct18	PL	0.70 ± 0.05	—	40.6
Oct18	EW	0.70 ± 0.05	—	5.3
Oct18	ΔH25	0.46 ± 0.05	—	10.9
Oct18	ΔH50	0.52 ± 0.05	—	43.5
Oct18	ΔH75	0.28 ± 0.05	—	—
Oct18	ΔPL	0.47 ± 0.05	—	46.8
Oct18	ΔEW	0.63 ± 0.05	—	18.0
Nov19	H25	0.73 ± 0.05	—	20.3
Nov19	H50	0.72 ± 0.05	—	41.6
Nov19	H75	0.68 ± 0.05	—	31.2
Nov19	PL	0.69 ± 0.05	—	26.4
Nov19	EW	0.72 ± 0.05	—	21.4
Nov19	ΔH25	0.56 ± 0.05	—	16.1
Nov19	ΔH50	0.54 ± 0.05	—	15.2
Nov19	ΔH75	0.43 ± 0.05	39.7	—
Nov19	ΔPL	0.51 ± 0.05	31.0	21.7
Nov19	ΔEW	0.68 ± 0.05	—	11.9

Data are shown for phenotypes height at 25%, 50%, and 75% of the body (H25, H50, and H75, respectively), PL, EW, and net gains traits associated (ΔH25, ΔH50, ΔH74, ΔPL, and ΔEW, respectively) in November 2017 (Nov17), October 2018 (Oct18), and November 2019 (Nov19).

### Future directions

In this study, the combination of QTL mapping and GWAS enabled the identification of genomic regions that control growth in a large captive trevally breeding population. From a farming perspective, parameters such as stocking density (Irwin *et al.* 1999) or feed availability and quality (Holm *et al.* 1990) can be carefully managed to accelerate fish growth in a land-based aquaculture facility. However, when aiming to develop new species for aquaculture, it is fundamental to understand the genetic architecture of commercially important traits that can potentially be enhanced through selective breeding. The findings of this study provide a useful framework for determining the genetic basis for growth traits in trevally. The identification of multiple QTLs through QTL mapping and genetic markers commonly involved in growth-related traits via GWAS represents an essential step for the implementation of a molecular breeding program for trevally. With a highly polygenic trait such as growth, genomic selection might be the most effective strategy to improve economic traits in this species. With this objective in mind, we are in the process of developing a medium-density SNP array to be used for the routine screening of the trevally breeding population (though note that such an SNP chip would also be useful for screening wild populations). Additionally, fine mapping, confirmation, and annotation of relevant regions identified in this study will bring a deeper understanding of the genetic architecture of growth in this species, and possibly also closely related teleost species.

## Data availability

Supplementary Table 1 contains a list of SNP-trait associations detected with GWAS for height at 25%, 50%, and 75% of the PL (H25, H50, and H75 respectively), PL, EW, and net gain traits (ΔH25, ΔH50, ΔH75, ΔPL, ΔEW) in November 2017 (Nov17), October 2018 (Oct18), and November 2019 (Nov19). For each SNP, the table shows the physical position on the LG, the *P*-value, the MAF, the FDR-adjusted *P*-value, and the percentage of variance explained (PVE).

Trevally (araara) are a taonga (treasured) species to Māori, the Indigenous people of Aotearoa New Zealand. All genomic data obtained from taonga species have whakapapa (genealogy that includes people, plants and animals, mountains, rivers, and winds) and are therefore taonga in their own right. These data are tapu (sacred) and tikanga (customary practices, protocols, and ethics) determine how people interact with these data. Thus, all the genomic data have been deposited in a managed repository that controls access. Raw and analyzed data are available through the Genomics Aotearoa data repository at https://repo.data.nesi.org.nz/. This was done to recognize Māori as important partners in science and innovation and as intergenerational guardians of significant natural resources and indigenous knowledge.

Supplemental material is available at *G3* online.

## Supplementary Material

jkac016_Supplementary_Data
